# The first two decades of CREB-memory research: data for philosophy of neuroscience

**DOI:** 10.3934/Neuroscience.2021017

**Published:** 2021-02-22

**Authors:** John Bickle

**Affiliations:** Department of Philosophy, Mississippi State University; Department of Neurobiology and Anatomical Sciences, University of Mississippi Medical Center. USA

**Keywords:** cyclic adenosine monophosphate response element-binding protein (CREB), memory consolidation, memory allocation, ruthless reductionism, mechanism

## Abstract

I recount some landmark discoveries that initially confirmed the cyclic AMP response element-binding (CREB) protein-memory consolidation and allocation linkages. This work constitutes one of the successes of the field of Molecular and Cellular Cognition (MCC) but is also of interest to philosophers of neuroscience. Two approaches, “mechanism” and “ruthless reductionism”, claim to account for this case, yet these accounts differ in one crucial way. I explain this difference and argue that both the experiment designs and discussions of these discoveries by MCC scientists better fit the ruthless reductionist's account. This conclusion leads to further philosophical discussion about how discoveries in cellular/molecular neurobiology integrate with systems neuroscience findings.

## 1. Introduction

Neurobiologist Yadin Dudai opens the entry on “CREB” in his handbook on memory concepts by remarking: “CREB is one of the most commonly used acronyms in neurobiology these days, and also one of the few words in the jargon of molecular biology that even experimental psychologists and computational neuroscientists might have encountered. And if they didn't, they should. Because the more we advance our knowledge in molecular neurobiology, the more we realize that CREB plays a pivotal role in the response of neurons to external stimuli” [1].

**Figure 1.**
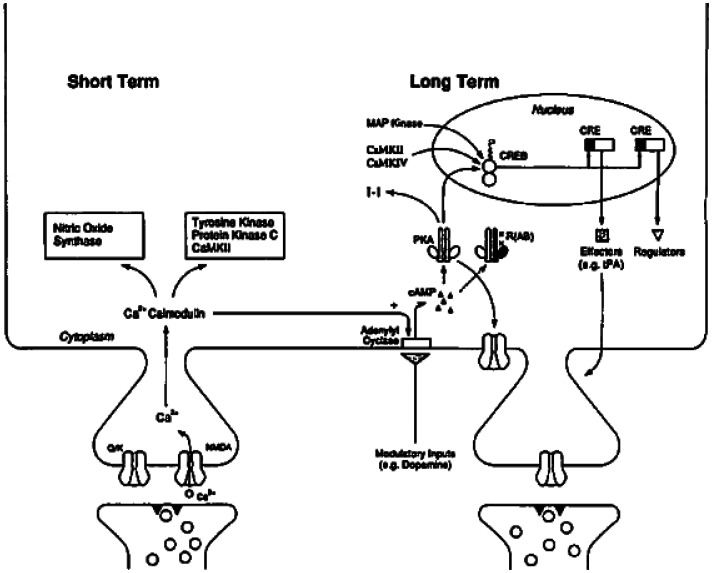
“A Molecular Model for the Consolidation of the Late Phase of LTP and Hippocampus-Based Long-Term Memory”. Reprinted with permission from [13].

“CREB” is the acronym for cyclic adenosine monophosphate (cAMP) response element-binding protein, a gene transcription factor that binds to specific DNA sequences (called cAMP response elements, or CREs) on the control region of various genes to influence their expression. CREB is activated by phosphorylation, the attachment of a phosphate group (PO_3_^−^) to various sites on the inactive protein. Phosphorylated CREB dimerizes with other CREB binding proteins to affect gene transcription. Some phosphorylated CREB isoforms^1^ are transcriptional activators, initiating activity of the messenger (m-) RNA polymerase docked at the gene's promoter region to begin the process of mRNA production. Other CREB isoforms are transcriptional repressors, inhibit gene transcription.^2^ (Gene transcription, with which CREB is exclusively involved, is only the initial step in mRNA production, which in turn is only one part of a subsequent multi-step process of gene translation, the synthesis of protein products.) CREB-activated gene transcription is an intermediate step in numerous intracellular signaling pathways, one of which begins with a rise in the amount of the second messenger cAMP inside the cell. In neurons, one way that intra-cellular cAMP levels rise is in response to activated biogenic amine-responding receptors (serotonin in invertebrates, dopamine in vertebrates). Neurotransmitter binding, released from other neurons synapsing on the affected neuron, activates the attached intracellular g-protein component of these receptors, which in turn primes adenylyl cyclase molecules to convert adenosine triphosphate molecules into cAMP. (See Figure 1 below; I will say more about this entire pathway as we proceed.)

Dudai offered this assessment a decade after CREB was first implicated as part of a molecular mechanism for memory consolidation in mammals. In the next section I recount some of the history of this fascinating episode in turn-of-the-21^st^-century neurobiology and behavioral neuroscience. In addition to its inherent scientific interest and importance, this case is philosophically interesting because the CREB-memory linkage has been embraced by two prominent yet contrasting accounts of the practices and goals of neuroscience, “mechanism” and “ruthless reductionism”. Some of the details of CREB's linkage with memory processes on the lab bench are helpful towards evaluating these competing philosophies of neuroscience. This evaluation in turn leads to deeper reflections into prospects for integrating discoveries from the many fields that comprise this interdisciplinary scientific endeavor. Despite hopeful rhetoric, genuine integration across the neurosciences remains scarce, and this for reasons I will suggest in the final section.

## 2. CREB and memory: the first twenty years of experimental investigations^3^

What does molecular biology have to do with memory? Since Hasker Davis and Larry Squire's [2] much-cited review essay from more than three-and-a-half decades ago, learning and memory researchers have known that while the initial acquisition of memory does not depend on new protein synthesis in neurons, the formation of long-term memories does. The conversion of short-term forms of memory into more stable, less easily disrupted and enduring long-term forms is known as the “consolidation” phase [3]. Davis and Squire reviewed numerous experiments in which a general protein synthesis inhibitor (such as anisomycin) was administered systemically to animals in vivo during various phases of learning and memory tasks. Based on the timing of administration, new protein synthesis was shown to play no role in initial learning or short-term memory recall, but was crucially involved in long-term memory encoding, and thus in the consolidation phase. However, since these inhibitors temporarily shut down all protein synthesis in the organism at the time of administration, and not gene transcription, they could not be used to investigate the transcriptional components or mechanisms involved in memory consolidation.

A study using invertebrates first suggested a role for CREB in the molecular processes specific to memory consolidation. Pramod Dash, in Eric Kandel's lab, led a team that injected oligonucleotides containing CREs into cultured sensory neurons from the sea slug *Aplysia californica* [4]. Oligonucleotides are short sequences of nucleotide residues (the residues that conjoin to make up entire DNA or RNA molecules) that trap specific proteins, in this case CREB-like proteins, and thereby block the proteins' usual effects. Dash et al.'s procedure inhibited serotonin-induced long-term increases in synaptic strength between *Aplysia* sensory and motor neurons, while leaving short-term synaptic facilitation unaffected.

Could a role for CREB be demonstrated in mammalian memory consolidation? Circumstantial evidence suggested that might be possible. Michael Greenberg's laboratory had established that CREB was activated as a gene transcription factor in mammalian neurons by two intracellular second messengers, calcium ions (Ca^2+^) and cAMP; and that CREB functioned as a substrate for depolarization-activated calmodulin kinase II (CaMKII) and protein kinase A [5]. In 1992, in Susumu Tonegawa's lab, Alcino Silva led a team that first succeeded in using a gene targeting technique to “knock out” a single gene in mammals and show an effect on behavioral measures of memory in the mutant mice [6],[7]. Silva and colleagues knocked out the gene for the α-isoform of CaMKII. Their technique knocked out the gene at the embryonic stem cell stage of development before cell differentiation, so all cells in the mutant mices' bodies, including neurons, lacked a functional gene for α-CaMKII. Homozygous mutants showed deficient long-term potentiation (LTP) in hippocampus neurons compared to wild-type littermate control mice. Mutants were also deficient in learning hippocampus-dependent behavioral memory tasks (i.e., contextual conditioning and the Morris water maze). These two papers were the first to introduce gene targeting techniques in mammals into the neurophysiology of LTP and the behavioral neuroscience of memory. (These techniques had been developed for mammals a decade earlier by developmental biologists.) With gene targeting techniques now demonstrably applicable to mammalian neuroscience, the new field of “molecular and cellular cognition” (MCC) was launched.^4^

Collectively, these early results suggested that a logical next step would be to knock out the gene for CREB and investigate mutants for deficient performance on rodent long-term memory tasks. After finishing his post-doc with Tonegawa, Silva started his own lab at Cold Spring Harbor National Laboratories (CSHL). In one of those remarkably serendipitous coincidences on which so many episodes of scientific progress turn, Günther Schütz, a molecular biologist from Heidelberg, Germany, was attending symposia at CSHL to present his research on the role of CREB in liver function. Schütz's lab had developed a homozygous CREB α/δ-isoform knock-out mouse. They provided mutants and wild-type littermate controls to Silva's lab for neurophysiological and behavioral testing.^5^

Using Schütz's mice, a team in Silva's lab led by Roussoudan Bourtchouladze showed intact early phase, but deficient late phase LTP in mutant hippocampus slices, as compared to wild-type littermate control mice [10]. They also showed intact mutant behavioral performance on a variety of initial and short-term memory tasks, but deficient behavioral performance on long-term versions of these same tasks, also compared to wild-type controls. Subsequent MCC research in Silva's and other labs found this same pattern of results on other rodent memory tasks in homozygous CREB α/δ-knock-out mice, including conditioned taste aversion, object recognition, and social recognition memory. (See [11] for a review of these experiments and results). Based on these many findings, CREB's role as a key molecule in a mechanism of memory consolidation became widely acknowledged among MCC researchers.

CREB's basic molecular biology was likewise becoming clarified. Besides the g-protein-coupled serotonin/dopamine-responding receptors → adenylyl cyclase → cAMP cascade, at least three other intracellular signaling pathways were shown to initiate CREB phosphorylation and activation as a transcription factor.^6^ So a CREB knock-out mouse alone could not determine which of these specific intracellular signaling pathways was responsible for CREB's role in memory consolidation. Initial attempts to knock out PKA to implicate the cascade involving it proved inconclusive. A team led by Ted Abel in Kandel's lab instead turned to the transgenic approach, by which a cloned gene is inserted into an organism at the embryonic stem cell stage of development (before cell differentiation has occurred) [13]. The extra copy (or copies) of that inserted transgene is thus present in every cell of the developed animal's body, but the transgene is attached to a specifically engineered promoter region. So the transgene is only transcribed, and the extra protein subsequently only translated and synthesized, in cells that have enough of the promoter molecule present to bind to the mRNA polymerase and turn on transgene expression.^7^ In Abel et al.'s case, the cloned transgene they inserted was for the regulatory (R) subunits of the PKA molecule. They coupled the PKA R transgene with a CaMKII promoter region, which limited transgene expression mostly to forebrain regions. PKA R subunits bind to the PKA protein's catalytic subunits, keeping the PKA protein in an inactive state. cAMP molecules bind to the R subunits, causing them to fall away from the catalytic subunits and enabling the catalytic subunits to phosphorylate target proteins. Catalytic PKA subunit-CREB interaction in vertebrates is complex and indirect, through activation of calcium/calmodulin-dependent kinase type IV or mitogen-activated protein kinases which directly phosphorylate CREB. But the outcome in the PKA R transgenic mutants is decreased phosphorylated CREB proteins in the neurons' nucleus to drive new gene transcription. In forebrain neurons of the PKA R transgenic mice, in which the PKA R transgene was expressed by the activation of its CaMKII promoter region, enough PKA R subunits should be available in the cytoplasm to re-bind any PKA catalytic subunits freed via the rise in cAMP, and so block this early step in the intracellular signaling pathway to phosphorylating CREB. This should in turn block subsequent LTP in these neurons and memory consolidation in hippocampus-dependent tasks, if the cAMP → PKA pathway for activating CREB is a mechanism for these effects.

These were exactly the results that Abel et al. found [13]. Early-phase LTP was normal in PKA R-transgenic hippocampus and cortical tissues slices (compared to wild-type littermate controls); but late-phase LTP declined after 40 minutes, then further declined to near baseline response rate by 120 minutes, far more quickly than wild-type controls. Behaviorally, in the Morris water maze the PKA R transgenic mice found the hidden platform at a rate comparable to control mice. But on a more sensitive probe trial on training day 15, when the escape platform was removed, PKA transgenics spent significantly less time in the quadrant where the platform had been located during all previous training episodes and crossed the training location of the platform significantly fewer times, than did wild-type controls. Most interestingly, Abel et al. trained animals on a combined contextual conditioning-fear conditioning task [13]. The first task is hippocampus-dependent; the second is amygdala-dependent. PKA R transgenic mutants matched control mice performance on learning, short-term, and long-term versions of the fear conditioning task. They also matched control performance on the learning and short-term version of the contextual conditioning task, but were significantly impaired on the long-term version. (Abel et al. suggest that difference results from “perhaps lower levels of R (A, B) transgene expression in the amygdala” [13]). Integrated with the earlier CREB studies, these results showed that the intracellular signaling pathway initiated by the active biogenic amine g-protein coupled receptor → rise in intracellular cAMP signaling pathway for phosphorylating CREB was a key mechanism in LTP and memory consolidation.

Combining all of these initial experimental results, Abel et al. sketch “a framework for a molecular understanding of the consolidation of explicit long-term memory” [13] (See Figure 1). This CREB-modulated post-synaptic causal pathway for mammalian LTP and memory consolidation has since become “textbook” neurobiology, with numerous additional molecular components added to the account (see e.g., [14] chapters 7 and 8). In a recent review Alberini and Kandel note numerous additional complex transcriptional events that have subsequently been discovered to be required for LTP and memory consolidation, but still refer to phosphorylated CREB as “essential ... for memory consolidation and reconsolidation” [15].

While there is still no consensus as to what are the critical downstream effectors of CREB activation for LTP or memory consolidation, evidence has been found for the movement of α-amino-3-hydroxl-5-methyl-4-isoxazoproprionic acid (AMPA) receptors from both intracellular storage pools and extrasynaptic sites to synaptic sites in the postsynaptic density. AMPA receptors, activated by the neurotransmitter glutamate, are the principal channels for sodium ion (Na^+^) influx into post-synaptic neurons, generating excitatory post-synaptic potentials, and in turn action potentials (“spikes”). The result of increased AMPA receptors at synaptic sites is specific post-synaptic neurons that generate stronger excitatory post-synaptic potentials, and hence are more likely to generate action potentials, to later excitatory neurotransmitter release from the same pre-synaptic cells, for extended periods of time, with this synaptic plasticity now linked to performance on behavioral measures of memory consolidation. As yet, however, no evidence exists that these AMPA receptor movements require CREB transcription.

Less than a decade after CREB's role in memory consolidation was first explored, CREB activity in another aspect of mammalian memory was discovered. Sheena Josselyn in Mike Davis's laboratory used gene insertion via virus vectors to show that overexpression of CREB in the rat amygdala in vivo enhanced behavioral measures of tone-fear conditioning [16]. Interestingly, the virus that Josselyn used to insert the extra CREB transgene infected only about 15% of lateral amygdala (LA) neurons. Previous electrophysiological studies had revealed that an episode of fear conditioning training to a specific tone activates only about 25% of lateral amygdala neurons. So if increased CREB activity at time of training had no effect on a neuron's probability of being recruited into the memory trace for a specific tone-shock association, slightly less than 4% (0.15 × 0.25) of LA neurons would be expected to have received the extra copy of the CREB transgene, and so show the experimentally manipulated higher CREB activity level, and have been recruited into a specific tone-shock memory trace. But might higher levels of CREB activity at time of training somehow bias specific LA neurons for recruitment into the engram for a specific tone-fear association memory?

Working in Silva's laboratory, Josselyn first reproduced in mice the CREB results with virus vectors she had obtained in rats. Increasing levels of activated CREB protein via virus vector insertion into mouse LA enhanced fear conditioning performance, while only infecting approximately 15% of neurons [17]. To test whether the CREB activity-enhanced neurons were more likely to be recruited into the engram for a specific tone-shock memory, Josselyn used a technique developed by John Guzowski, in collaboration with Carol Barnes and Paul Worley [18]. Guzowski used florescent in situ hybridization to show that in recently activated neurons, mRNAs for the activity-related cytoskeletal associated protein (ARC) accumulates in cell nuclei and cytoplasm at different times after the high neuronal activity. With ARC mRNAs fluorescently labeled using Guzowski's technique, their presence in a cell's nucleus could be used to identify a cell that had been highly active at time of recall performance on the memory task, thereby identifying that neuron as a component of the engram for that specific tone-shock memory. In tissue slices taken immediately from treated behaving mice brains, the nuclei of recently active cells will fluoresce against the dark background of less active cells. Thus, if ARC fluorescence is found preferentially in the nuclei of the CREB activity-enhanced cells during retrieval, this would indicate that allocation of neurons into specific memory traces is biased toward cells with higher levels of CREB activity at time of memory acquisition.

Josselyn inserted the CREB transgene, to which she attached the gene for Green Fluorescent Protein (GFP), in mouse LA. GFP expression, coupled in these mice to CREB transgene expression, causes neurons in tissue slices to glow green under standard light microscopy. She trained mutant mice and wild-type littermate controls on amygdala-dependent tone-fear conditioning. Images from GFP slices were compared, neuron for neuron with images from ARC fluorescent slices, to see how many neurons overlapped, i.e., had both high CREB activity driven by the transgene insertion at time of learning and high recent activity during the retrieval phase of a specific tone-shock conditioning revealed by in situ hybridization of nucleus ARC mRNAs. CREB-enhanced cells were three to four times more likely than non-infected neurons to show ARC expression, indicating high activity during specific memory retrieval. In contrast, decreasing the levels of CREB in neurons via virus insertion of a mutated CREB gene that interfered with CREB function had the opposite effect. Cells transfected with mutant CREB were only one-twelfth as likely to express nucleus ARC mRNA at retrieval time than non-infected neurons. Numerous control experiments were necessary to interpret these findings, but in the end Han et al. conclude “our results suggest a competitive model of memory formation, in which eligible neurons are selected to participate in a memory trace as a function of their relative CREB activity at the time of learning” [17]. Beyond its well-established role as a component in a molecular mechanism of memory consolidation, CREB also appears to function in memory allocation, i.e., the allocation of specific memories to activation of specific neurons.

This role for high CREB activation in the allocation of specific tone-fear memories was further elaborated by subsequent studies. One example from Silva's lab comes from a team led by Yu Zhou, who attached an engineered gene from Drosophila for an allatostatin receptor, insertable and expressible in mammalian neurons [19]. Activation of that engineered receptor via allatostatin peptide dampens neuron activity. Attaching that engineered gene to the CREB transgene and inserting the combination via a virus vector would enable the allocation of a specific tone-fear memory preferentially to infected LA neurons, but then allow experimenters to dampen down activity in exactly those neurons in later retrieval tests via allatostatin peptide administration. This process indeed attenuated memory recall performance in the behaving mice. But memory for the tone-shock association remained or later returned to normal in these mice on retrieval tests without allatostatin peptide administration.

Another follow-up study came from Josselyn's lab. Jin-Hee Han led a team that instead killed the transgenic CREB-enhanced LA neurons after their preferential recruitment into a specific tone-shock memory [20]. They used an inducible diphtheria-toxin strategy to kill specifically the transgenic CREB-enhanced neurons after their preferential activation by fear memory expression. Selectively deleting the LA neurons overexpressing CREB after learning blocked subsequent expression of the fear memory; deleting a similar portion of randomly selected LA neurons via the diphtheria-toxin had no effect on subsequent fear memory expression. The memory loss in the first group was permanent, suggesting that the fear memory was erased. Han et al. conclude that their results “establish a causal link between a specific neuronal subpopulation and memory expression, thereby identifying critical neurons within the memory trace” [20]. Level of CREB activation was central to the specific neurons to which a given memory was allocated.

Even this brief history of the CREB-memory linkage elaborates Dudai's [1] quotation at the outset of this paper. These experiments and results have been among the most celebrated in the now three-decade history of MCC. But this case also prompts philosophical reflection on neuroscience-in-practice, to which I turn next.

## 3. Mechanism versus ruthless reductionism in recent philosophy of neuroscience

Two contrasting accounts in recent philosophy of neuroscience, “mechanism” and “ruthless reductionism”, both claim to incorporate findings such as the CREB-memory consolidation and allocation linkages. Not surprisingly, these two approaches agree about much concerning neuroscience's practices and products. Both accounts propose to start philosophical reflection from actual neuroscience practices, and so both recognize that philosophical assertions made about neuroscience must answer to what actually happens in labs and gets published in papers. Both accounts recognize the centrality of manipulation or intervention experiments, and both recognize a related variety of distinct types of such experiments. Both accounts view neuroscience as focused primarily, or at least centrally on discovering mechanisms. Both acknowledge explicitly that the work of cellular and molecular neuroscientists makes important contributions to explaining cognitive phenomena. This last point of agreement is especially important because it rejects the tendency of many other philosophers to focus exclusively on cognitive and systems neuroscience as the only fields in the discipline that are of philosophical relevance. Both mechanists and ruthless reductionists insist that such a limited focus, though popular, leads to a grossly distorted picture of neuroscience as a scientific endeavor.^8^

**Figure 2.**
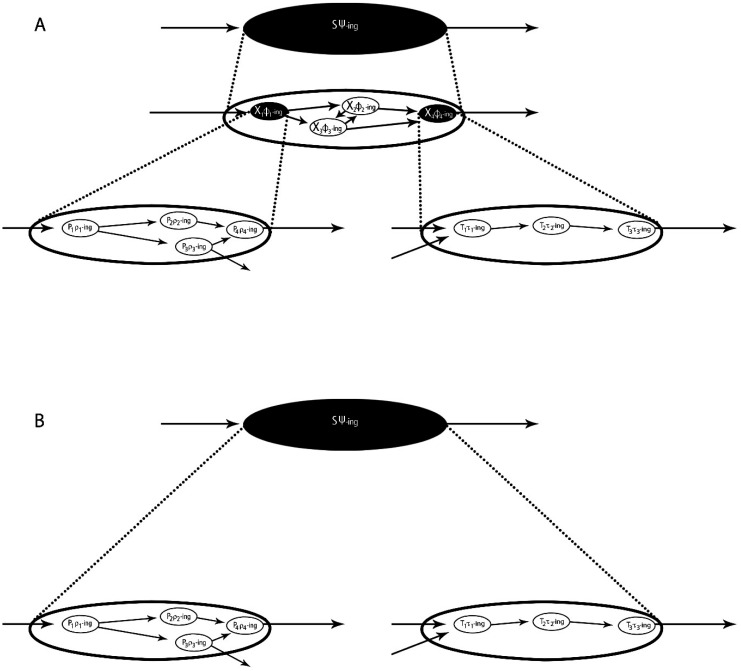
A. Mechanists' “Nested Hierarchy of Mechanisms-Within-Mechanisms” Account, with only one intermediate layer of mechanisms pictured. B. Ruthless reductionists' “Direct Mind-to-Molecular Pathways Linkages” Account. The top and bottom levels on both illustrations are supposed to be identical. See text for discussion. Figure 2A, B reprinted from [27] with permission from Cognitive Science Society, Inc. Original figure and adaptation by Pamela Speh.

These points of agreement are found throughout the most cited writings of mechanists [22]–[25] and ruthless reductionists [8],[11],[26]. Yet one crucial difference between the two accounts persists: the mechanists' advocacy of “nested hierarchies of mechanisms-within-mechanisms” versus the ruthless reductionists' advocacy of “direct mind-to-molecular pathways linkages.” Figure 2 illustrates this difference, although for brevity only a single intermediate level of mechanism is pictured in Figure 2A. The top level of both figures reflects the system, e.g., the behaving rodent, engaging in some behavior that operationalizes a cognitive function, e.g., traversing a Morris water maze as an indicator of spatial memory. The bottom level of both figures reflects the inter- and intra-neuronal molecular cascades, which in this case would include CREB activation and subsequent gene expression in hippocampus and cortical neurons, embedded in the entire set of molecular interactions (neurotransmitter release, receptor binding, intracellular signaling pathways activity) stretching back to the animal's activated sensory receptors and out to its motor periphery. The intermediate layer pictured in Figure 2A might be, e.g., hippocampus place cells forming spatial maps, although in the classic mechanist diagram of rodent spatial memory, two intermediate layers of mechanisms-within-mechanisms are illustrated, with the level just above the molecular cascade being the individual hippocampus neurons undergoing LTP [24]. The point is, for mechanists, the lowest level diagrammed is NOT a mechanism of the highest level diagrammed, e.g., of the rodent navigating the water maze. The lowest level instead is a mechanism *of the next level up*, e.g., of the individual hippocampus neurons undergoing LTP. And the latter is the mechanism *of the next level up*, and so on, until finally the penultimate level from the top is the mechanism for the system's behavior, e.g., the rat navigating the water maze. The ruthless reductionist, on the other hand, is perfectly willing to claim that the molecular mechanisms are *a mechanism of the system's behavior*, so long as the right collection of integrated intervention experiments confirm this.

For those who wonder whether this distinction actually constitutes a difference, consider that mechanists make a big deal over it. According to Bechtel the mechanist account fully accommodates molecular neurobiology, but also locates those mechanisms at their appropriate level in the hierarchy of mechanisms-within-mechanisms constituting the cognitive system [23]. Those interacting molecules are *not* mechanisms of the organism's (system's) *behavior*. Instead, they are mechanisms of functioning individual neurons, conducting action potentials and transmitting activity to other neurons across shared chemical synapses. Individual neurons in turn are mechanisms of functioning neural circuitries or networks, and on up the mechanism hierarchy ultimately to the behaving system. According to Bechtel, by locating each mechanism at its proper level in the nested hierarchy, the mechanist account does not falsely accord the molecular mechanisms any special epistemic or explanatory role or privilege. They are just another level of mechanisms hierarchically organized to constitute the cognitive system. Bechtel insists that this recognition accords to mechanist philosophy of neuroscience an epistemic advantage over ruthless reductionist: “What is gained by paying attention to each level of organization identified in the course of decomposition is an understanding of the variety of roles that must be performed in producing the overall phenomena” [23]. By focusing exclusively on intra- and intercellular molecular mechanisms, ruthless reductionists risk overestimating the causal importance of one or a few of these components. So ruthless reductionism “fails to consider how those sub-components are related to others in the realization of the phenomena in question... One serious shortcoming of ignoring these other factors is the propensity to ascribe too much to the factors that are considered” [23].

Ignoring intermediate levels of mechanisms-within-mechanisms and advocating for direct mind-to-molecular pathways linkages reminds Bechtel of a similar “ruthlessly reductive” approach that in retrospect wreaked havoc in genetics: The legacy of focusing on genes as responsible for traits, ignoring the variety of factors involved in the regulation and expression of genes, illustrates the risks involved. Researchers often claimed success in explaining biological traits as soon as they identified a responsible gene, failing to recognize that often many factors, including other genes as well as environmental processes, are required to generate the trait [23]. Mechanists' recognition of nested hierarchies of mechanisms-within-mechanisms will not be tempted toward making this mistake.

Another leading mechanist, Carl Craver, argues that recognizing the nested hierarchies of mechanisms-within-mechanisms permits mechanists to identify the specific components, activities, and organization that are *relevant* for the specific cognitive operation under investigation. On the other hand, Craver insists, pursuing ruthlessly reductionist neuroscience, and thus ignoring top-down constraints imposed on lower level investigations by fields of neuroscience that address higher level components in the nested hierarchy, runs serious risk of losing sight of the boundaries of the mechanisms for specific cognitive functions, and thereby including in one's explanation more processes than just the relevant ones. In this context Craver quotes with approval philosopher Phillip Kitcher's famous quip about reductionism (ruthless or otherwise): “a world viewed only at the fundamental level would be a world of gory details unfiltered by a higher level perspective” [24]. So it's not just ruthless reductionists who insist that these accounts differs in at least this one significant respect (see [26], sec. 6 and below); mechanists concur.^9^

Since both mechanists and ruthless reductionists agree that philosophy should “start with neuroscience,” it is fair to ask, how did the neuroscientists who forged the CREB-memory consolidation and allocation linkages (i) design their experiments and (ii) describe their results? Did they do so more in keeping with the mechanists' stress on the proper place of molecular mechanisms within a nested hierarchy, or more in keeping with the ruthless reductionists' stress on finding direct linkages of cognitive functions to molecular processes? Do their experiments and their discussions look more like Figure 2A or B?

One source of data for answering such questions are scientists' discussions in landmark experimental publications. Careful attention to these discussions is part of a method that Silva, Landreth and Bickle call “metascience” (or “science of science”, “S2”) [8]. There are limitations to this approach. Journal publications are highly “processed” scientific products. They typically do not reflect the day-to-day laboratory practices that generated either the experiments or the data reported in the paper. The focus of the journal in which the manuscripts are published can impact what scientists report and what they discuss about their results. It is incumbent on the practitioner of metascience to have first-hand experiences of the research practices in the field he or she is investigating, as an active participant in the research, not simply as an anthropologist-style “outside observer”. These experiences must include the activity of writing scientific publications, so the metascientist can know first-hand how the process of transforming experiments and results into published scientific papers works. Only then can the metascientist reasonably draw inferences—and defeasible inferences at that—about actual practices from careful readings of publications. Sometimes supplemental materials give a more accurate reflection of the motivations that generated the specific experiments and data reported than do the publications themselves, and should be investigated when available. Sometimes one might use structured interviews with the scientists.^10^ Even at its most effective, the metascientific approach yields defeasible conclusions. It cannot purport for or claim deductive validity for its arguments. But if philosophy of neuroscience is to “start with neuroscience,” as both mechanists and ruthless reductionists insist, investigating what neuroscientists do, including what they write, is methodologically inescapable.^11^

Without question there are neuroscientists who design experiments, construct computational models, and discuss their results in language reflecting mechanists' image of nested hierarchies of mechanisms-within-mechanisms. Bechtel presents numerous examples [23]. But the brief history presented in the previous section shows that other neuroscientists pursue direct mind-to-molecular pathway, ruthlessly reductive experiment designs. Some component of an intra- or inter-cellular signaling pathway is manipulated and the effects *on the overall behaving system (organism)* are monitored. Intermediate levels of nested mechanisms-within-mechanisms are not direct targets of the experimental manipulations or interventions. Of course, the experimental manipulations and interventions of these molecular pathways will have measurable effects on intermediate-level mechanisms. If manipulating CREB or PKA molecules in specific neurons produces effects on the organism's behavior, doing so will also produce effects in specific neural circuits and regions, and on the plasticity of the individual neurons that compose them. That is a simple fact of componency organization. But in the research sketched in the previous section, those intermediate-level effects were not the ultimate targets of the researchers. Typically those researchers did measure cell-physiological effects of their interventions, such as LTP in the specific neurons they intervened into. But these measurements were just a step on the way to their ultimate aim, namely, of showing that manipulation of a single component of a known molecular causal pathway in vivo had measurable effects *on behavioral indicators* for a specific cognitive function, e.g., ones for explicit memory in rodents. In reference to Figure 2B above, a “tweak” to a component in the causal pathway illustrated on the bottom level was shown directly to affect *system (organism) behavior* at the top level. That is what Bourtchouladze et al. showed first with Schütz's CREB knock-out mice [10], what Abel et al. showed with their PKA R transgenics [13], and what Han et al., Zhou et al., and Han et al., (2009) showed for the role of high intraneuronal CREB activity in the allocation of specific tone-fear memories to activity in those specific lateral amygdala neurons [17]–[20].

Furthermore, these scientists speak explicitly of having discovered *molecular mechanisms of cognitive functions*, without appealing to any stepwise restriction of mechanisms only to the next layer up in some nested hierarchy. In their original CaMKII mutation study, Silva, Tonegawa and colleagues took their in vivo behavioral data as indicating “that *α-CaMKII* has a prominent role *in spatial learning*” [7]. They insisted that their work “demonstrates that *a mutation in a known gene* is linked to *a specific mammalian learning deficit*, and indicates that *single genetic changes* can have a selective but drastic impact *on learning and memory*” [7]. These authors do not claim that their genetic manipulation and resulting mutated protein affects only spiking activity in individual neurons, which in turn affects only activity in some network circuitry, as the mechanist's nested hierarchy account would counsel. Instead, they insist that *the gene/molecule experimentally intervened upon* affected *the cognitive function itself*, operationalized behaviorally for experimental testing. One *can* interpret these claims along mechanistic lines, by insisting that these scientists were simply leaving the hierarchy of intervening intermediate-level mechanisms unstated, perhaps as enthymemes. But doing this requires putting additional words in these scientists' published writings. The scientists themselves don't include those additional words. So recognizing the defeasibility of metascientific conclusions mentioned above, ruthless reductionism seems better to capture what these scientists both did and wrote about what they did.

Bourtchouladze and colleagues spoke similarly. Their results with CREB mutant mice “implicate *CREB-dependent transcription* in *mammalian long-term memory*” [10]. Their findings “identify *CREB* as *an important component of memory consolidation in mammals*” [10]. “Component” is a mechanism-word, but it is not being used in this passage to refer to some intermediate level of mechanisms between the molecular pathway involving CREB activity and the cognitive function. Rather, Bourchouladze et al. call CREB a “component” *of the cognitive function itself*, just as the ruthless reductionist pictures it (Figure 2B above). Abel and colleagues likewise evoked the ruthless reductionists' picture, making no mention of any intermediate levels of mechanisms. “*PKA* plays a role *in the consolidation of long-term memory*” [13]. “*The PKA pathway* is critically important *for the consolidation of short-term memory into protein synthesis dependent long-term memory*” [13]. “Our experiments ... provide a framework for *a molecular understanding* for *the consolidation of long-term explicit memory in mice*” [13]. Not for cellular activities, nor for network activities, but for *learning and memory itself*. That's what's pictured in Figure 2B above, not Figure 2A.

Does all this imply that these scientists advocate “eliminating” the intermediate levels between intra- and inter-neuronal molecular pathways and the behaving organism? No! This is one way that ubiquitous “levels” diagrams and talk misleads. One can acknowledge the neurons and the networks they form as components of the behaving system (organism). What successful experiments of the sort surveyed in section 1 do is link controlled experimental manipulations of a component of the molecular pathways directly to behavioral effects, obviating the *explanatory need* to evoke cell-biological or network phenomena in the mechanism explaining system behavior. Nothing more. For *explanatory purposes*, the molecular pathways within and between specific neurons suffice. None of this implies the nonexistence of these neurons or networks, nor of their explanatory usefulness for other data or purposes; only for their *explanatory dispensability* for the target cognitive phenomena operationalized behaviorally in the ways employed in the experiments described.

The “levels” metaphor is so ingrained in both science and philosophy of science that its demise is probably too much to hope for. But a better metaphor would be one of concentric organism-shaped blobs of coarser and finer grain, each one taking up the entire space of the behaving organism. The entire behaving system would be the concentric blob of coarsest grain, the networks or circuits of neurons the concentric next-finer blob, the entire collection of individual neurons comprising all these networks or circuits the next finer, and the entire collection of intra- and inter-neuronal molecular pathways the next finer still. No “ontological levels” require bridging on this picture; one and the same system is organized at coarser and finer grains, and our experimental technologies enable us to manipulate single components in the molecular pathways in order to evoke significant alterations in system (organism) behavior. Ironically, the “levels” metaphor, along with many of its current puzzles, trace back to medieval “levels of Being” discussions. Must our early-21^st^ century scientific image still rest on a metaphor grounded in theological disputes from more than eight centuries ago?

From a metascientific perspective, the scientists who initially linked CREB activity to memory consolidation and allocation designed their experiments and described their results more like ruthless reductionists than like mechanists. So ruthless reductionism is alive and well in the practices of quite a few neuroscientists. Mechanism's popularity in recent philosophy of neuroscience does not reflect a dominance in actual neuroscience-in-practice. But these philosophical-metascientific reflections raise other concerns. What implications might these differences in experimental practices and explanatory goals carry for unification and integration of results across the numerous disciplines that compose contemporary neuroscience?

## 4. “Can't we all just get along?”

I have argued that some landmark results from the first twenty years of MCC reflects ruthlessly reductive neuroscience practice. I have also assumed that other neuroscientists, including some MCC researchers, pursue work more in the fashion championed by mechanists. Ruthless reductionism and mechanism differ in at least one significant respect, as depicted in Figure 2 above. Some might wonder whether a coherent scientific endeavor can accommodate conflicting approaches like these. Surely there must be only one ultimately correct way to integrate cellular and molecular neuroscience with cognitive/systems neuroscience?

On the contrary. Why should neuroscientists have to speak in one voice about how to integrate results across fields? Neuroscience is an inherently interdisciplinary science. Neuroscientists come from a variety of scientific backgrounds, ranging from physics to chemistry to molecular biology, up to systems biology, experimental psychology, and cognitive science. The scientific intuitions and hunches about how best to address a given phenomenon will differ as widely as do neuroscientists' backgrounds and interests. Why expect “big picture” unity among such a motley crew, or hope that one philosophical approach will reflect the work of everybody involved in the enterprise? Instead, “why can't we all just get along”, and pursue whichever problems strike us as important and addressable, using whichever tools and experiment designs strike us as most promising, and executing experiments and discussing results in whatever way strikes us as most appropriate given our myriad scientific heritages? And simply accept that different neuroscientists will make different choices at each point?

Bechtel suggests something like this happily disunified picture [23]. He relates a brief history of the decade-and-a-half work that constituted the Neuroscience Research Program, (NRP) led by Francis Schmitt, which culminated in 1970 in the formation of the Society for Neuroscience. Although the stated goal of the NRP was to explain the human mind in terms of the brain, its principal scientists were biophysicists, biochemists, and molecular biologists. The closest scientist to a psychologist, or even a neurophysiologist, was a neurologist best known for his early-career work doing cortical ablation experiments in nonhuman primates. As Bechtel points out, this “primary endeavor for which the term *neuroscience* was employed indeed reflects the project of ruthless reductionism, a narrowing of focus to the molecular processes within individual neurons” [23]. As for the NRP's successor organization, the Society for Neuroscience, while it quickly welcomed systems and cognitive neuroscientists, “the predominant emphasis remains on cellular and molecular neuroscience: as Bickle has pointed out, it reflects the approach of the ruthless reductionist” [23]. On the other hand, the more recent emergence of cognitive and systems neuroscience as specific fields in their own right, the history of which Bechtel also sketches briefly [23], better reflects the mechanists' nested hierarchies of mechanisms-within-mechanisms account. Such researchers “are fully aware that the brain regions they study are composed of neurons and that processes such as the generation of action potentials and the altering of communication at synapses are crucial to the behavior of the brain regions” [23]. But those molecular components and their activities “are at a lower level of organization: operations at that lower level can explain how the components they study”—the circuits, the regions, the architectures—“perform their operations, but not how those operations together perform the overall cognitive task, which is their objective” [23]. And yet these two groups of scientists, with their own experiment tools, approaches, and explanatory goals, currently reside together harmoniously in the messy but thriving field we call “neuroscience”.

If Bechtel's historical narrative is accurate—and I have no reason to doubt that it is—then from a metascientific perspective contemporary neuroscience is both ruthlessly reductive *and* mechanistic. And no doubt a whole lot else: computationalist, evolutionary, dynamicist, extended/enactive... And there's no reason for it not to be all these things. “Highly interdisciplinary” implies highly variable, in experimental methods, research tools, designs, goals, explanatory practices, assumptions... In short, in all the ways that make up a given scientist's practices or “approach.” The philosophy of such a field should reflect this variance.

However, it is also important to realize that no field this varied can be tension-free. “Interdisciplinary” does not mean or imply “ecumenically cooperative”. In fact, “interdisciplinary” as understood by administrators—the picture of scientists from these various disciplines constantly mixing and matching up in joint research endeavors and adopting the tools and approaches of their fellows—is relatively rare. The physical locations of differently focused neuroscientists often differ. Cognitive and systems neuroscientists are often housed on academic campuses; cellular and molecular neuroscientists are often housed on medical campuses. Cognitive and systems neuroscientists in the United States often apply for research funding to the National Science Foundation and private research sources; cellular and molecular neuroscientists more often turn to the National Institutes for Health. In research projects involving neuroscientists from across these divides, as for example in drug discovery and development, the specific tasks of each participant are typically highly circumscribed and limited to their own areas of expertise. The Society for Neuroscience's official journal, *The Journal of Neuroscience*, currently divides each issue into five distinct areas, with different editors and reviewers for each, and authors typically choose the appropriate area for their submission: cellular/molecular; development/plasticity/repair; systems/circuits; behavioral/cognitive; and neurobiology of disease (http://www.jneurosci.org/). So despite the field's overall variety of approaches, neuroscience “on the hoof” maintains rather sharp and institutionalized divisions. A variety of flora blooms across the entire field, but that should not lead one to think that it is all one big, happy cross-fertilizing garden. At least in their day-to-day lab work, most neuroscientists spend time with others who are closely aligned with their own training and interests. This feature of actual neuroscience practice is a topic worth serious philosophical reflection. Perhaps “multidisciplinary” rather than “interdisciplinary” is a more apt descriptor for contemporary (and foreseeable?) “neuroscience-in-practice”? How much do these real-world restrictions limit our capacity to integrate neuroscientific results that span the molecular to the cognitive—a goal that to this day remains much more as happy rhetoric than concrete accomplishment? These reflections beg for further philosophical-cum-scientific investigations.

## Footnotes

1 Isoforms are structurally very similar proteins, originating from a single gene or gene family. The functions of different isoforms of the same protein can differ significantly, as in the case of CREB.

2 This discussion oversimplifies a very complex CREB molecular genetics. The context of the CRE binding sites and the regulation of the particular promoter also help determine whether CREB dimerization and binding activates or represses gene expression at that site.

3 My presentation of both the scientific details and the history in this section has been aided by very helpful remarks on a previous draft by Wayne Sossin.

4 See [8] for a survey of some landmark results of the first two decades (1992–2012) of MCC. Since 2000 there has been a scientific society, the Molecular and Cellular Cognition Society, with a worldwide membership, affiliates in Europe and Asia, and an annual meeting held each year in conjunction with the Society for Neuroscience annual meeting, along with affiliate meetings. See https://molcellcog.org/ (queried December 9, 2020).

5 Generating viable homologous CREB α/δ-knock-out mice turned out to be no easy task; A detailed account of the generation of Schütz's mutants is provided in [9]. The last sentence of [9] reads: “The relatively high abundance of CREB protein in brain ... and recent data implicating cAMP-inducing genes in long-term potentiation [citing [4], discussed above] and the maintenance of circadian rhythms make the CREB -/- mouse a useful model to study selective effects on neuronal function.” So it turned out to be, as we are about to see.

6 A much-cited review is [12].

7 These early transgenic techniques differ from today's CRISPR-cas9 technologies, as unlike the latter they afforded little control over where the transgene gets inserted into the DNA molecule.

8 For a review see [21].

9 Thanks to David Colaço who emphasized to me the importance of spelling out this difference between these two philosophies of neuroscience.

10 For an excellent recent example of how to do these structured interviews fruitfully and responsibly see [28].

11 I thank Wayne Sossin for raising concerns about this section in an earlier draft that required me to more clearly state the methods and limitations of metascience.
